# Benefits of Table Tennis for Children and Adolescents: A Narrative Review

**DOI:** 10.3390/children11080963

**Published:** 2024-08-10

**Authors:** Daniel González-Devesa, Miguel Adriano Sanchez-Lastra, Martín Pintos-Barreiro, Carlos Ayán-Pérez

**Affiliations:** 1 Well-Move Research Group, Galicia Sur Health Research Institute (IIS Galicia Sur), SERGAS-UVIGO, 36310 Vigo, Spaincayan@uvigo.es (C.A.-P.); 2 Departamento de Didácticas Especiáis, Universidade de Vigo, 36310 Vigo, Spain; 3 Facultad de Ciencias de la Educación Y del Deporte, Universidad de Vigo, Campus a Xunqueira, s/n, 36005 Pontevedra, Spain; martinpintosbarreiro@gmail.com

**Keywords:** coordination, disabilities, executive function, motor skills, table tennis

## Abstract

This study aims to review the scientific evidence regarding the effects of table tennis practice on children and adolescents. Studies were searched in three electronic databases (PubMed, Scopus, and SportDiscus) from their inception up to May 2024. The methodological quality of the included studies was assessed using the 10-point Physiotherapy Evidence Database (PEDro) and Methodological Index for Non-Randomized Studies (MINORS). A total of twelve studies were examined, with interventions involving children with intellectual disabilities, ADHD, DCD, ASD, and typically developing children. A variety of training programs were assessed over durations ranging from 6 weeks to 1 year in the studies included. Table tennis was shown to positively impact various domains, including executive function, motor skills, visual perception, graphomotor function, gross motor skills, coordination capacity, behavioral inhibition, and social behavior. Nonetheless, it is imperative to expand the number of studies on children and adolescents with diverse conditions to more comprehensively evaluate the benefits of table tennis for each specific condition.

## 1. Introduction

In today’s society, where insufficient physical activity and sedentary behaviour have led to an unhealthy body composition, with a tenfold increase in overweight and obesity rates over the past 40 years reaching epidemic proportions [1], it is crucial to promote the importance of regular physical activity in all its forms, especially during childhood and adolescence, as it fosters healthy growth and development [2]. Children who develop active lifestyle habits are more likely to maintain a healthier lifestyle in adulthood [3]. Regular exercise throughout life, starting from childhood and adolescence, contributes to a longer life with a higher quality of life by improving cardiometabolic and cognitive functions [4,5], and delaying the onset of many diseases [6,7,8].

Scientific consensus indicates that regular physical activity during childhood and adolescence is one of the most effective methods for improving the health of young people and adults. The development of motor skills and physical fitness in youth reduces the risk of metabolic and cardiovascular diseases, increases bone mineral density, reduces symptoms of depression [9], and enhances emotional, social, and cognitive well-being [10,11]. It also improves motor competence [12]. Additionally, there is evidence that motor skills improve healthy physical conditions in the medium and long term [13].

These findings have led public institutions to recognize the need to promote physical activity, particularly among children and adolescents. International organizations such as the World Health Organization (WHO) develop guidelines on PA and sedentary behaviours [14], targeting all age groups and those relevant to any society, sex, or culture. A specific chapter for children and adolescents (5 to 17 years old) recommends an average of 60 min of moderate-to-vigorous intensity of aerobic activity daily. This activity should be included in leisure activities, physical education in schools, commuting (walking, cycling, or other wheeled transport), or household chores. These recommendations are currently implemented across all administrative levels—governmental, regional, and municipal—as a social obligation and demand.

Among the options for exercise, table tennis (TT) stands out as a universal practice for children [15]. In this vein, Gatouillat et al. [16] found that racket sports, including TT, ranked among the most popular choices for boys, following team sports and combat sports, when analysing sports preferences among adolescents. Similarly, on a global scale, TT is considered the most popular racket sport and ranks second in terms of overall participation. Over 10–18 million players compete in numerous tournaments each year [17]. Indeed, the popularity of TT has constantly increased since it became an Olympic sport in the 1990s, reaching over 300 million practitioners worldwide [15].

This sport could be an effective strategy to motivate children and adolescents to engage in regular physical activity, potentially achieving optimal health outcomes and reducing sedentary behaviour [14].

Various authors have demonstrated the health benefits of TT. Pradas et al. [18] by comparing children who played TT recreationally with physically active children and found that those who played TT had less adipose tissue, indicating that TT has benefits in maintaining a healthier body composition. The same study concluded that TT helps to develop significant muscle mass and increases bone mineral content and density, particularly on the dominant side. According to Akramjonovich et al. [19], TT is a comprehensive sport that improves both upper and lower body muscles, enhances the cardiorespiratory system, and positively impacts motor skills and executive functions.

Given this information about TT, it is worthwhile reviewing the scientific evidence regarding the effects of its practice on children and adolescents. The obtained findings could provide valuable insights into the benefits of practicing this sport for this population.

## 2. Materials and Methods

This review was conducted in accordance with the Preferred Reporting Items for Systematic Reviews and Meta-Analyses (PRISMA) guidelines [20]. This review was registered with the Open Science Framework (OSF), https://doi.org/10.17605/OSF.IO/E3U2X (accessed on 26 July 2024). 

### 2.1. Search Strategy

A search strategy was developed to identify the maximum number of studies analyzing the effects of TT training on children and adolescents. This search was conducted in PubMed, Scopus, and SportDiscus databases from their inception up to May 2024, using the following keyword combinations and Boolean operators: “table tennis AND children”, “table tennis AND youth”, and “table tennis AND adolescents”.

Studies were excluded if they did not meet any of the following criteria: (1) studies that were not interventions; (2) interventions that did not have a specific TT group; (3) interventions that did not include participants aged ≤18 years; (4) studies written in languages other than Spanish, Portuguese or English.

### 2.2. Selection Process

Initially, a search was conducted by combining the keywords, and articles suggesting a TT intervention or including any of the aforementioned terms based on their title and abstract were selected.

The process began by classifying studies as “valid (a priori)” or “invalid” based on the previously mentioned selection criteria. Duplicate records were then removed. The full text of the studies whose title or abstract did not provide sufficient information for inclusion or exclusion was subsequently reviewed. After reading the full texts, studies that did not meet the selection criteria were discarded, resulting in a final set of studies classified as valid.

Finally, the references of the full-text articles and studies citing them were reviewed through Google Scholar. This secondary search identified additional articles that could be “valid (a priori)”, and the process was repeated until a final set of “valid” studies was obtained. In cases of uncertainty, another author was consulted.

Searches were independently conducted by one author and subsequently reviewed by a second author. In case of disagreement, a consensus was reached through the input of a third author.

### 2.3. Data Collection

The data extraction process involved organizing information from the selected studies into tables (Tables S1–S5). Each study was analyzed for: (1) authors and study design; (2) sample characteristics (sample size, dropouts, sample characteristics); (3) proposed intervention (program duration and intensity/volume/frequency); (4) variables studied (assessment tools); and (5) results obtained after the intervention.

### 2.4. Quality Appraisal

The quality appraisal was independently conducted by two authors, both holding doctorates in Physical Activity and Sports Sciences and possessing extensive experience in conducting reviews and meta-analyses. Input was sought from a third author in case of disagreement. 

The methodological quality of the included RCTs was rated using the 10-point Physiotherapy Evidence Database (PEDro) Scale. The evaluation was directly sourced from PEDro’s database for the studies already analysed. Studies were rated based on their quality as excellent (9–10), good (6–8), fair (4–5), and poor (<3) [21].

The Methodological Index for Non-Randomized Studies (MINORS) [22] was employed to assess the methodological quality of non-randomized studies. The MINORS instrument comprises 12 items for comparative studies and 8 items for non-comparative studies, each representing a distinct quality criterion. Items are scored as follows: 0 (not reported), 1 (reported but inadequate), and 2 (reported and adequate). The maximum possible score is 16 for non-comparative studies and 24 for comparative studies. For comparative studies, a MINORS score of 17 or higher was classified as high quality, while a score below 17 was considered low quality [23]. Non-comparative studies were deemed high quality if they achieved a score of 10 or higher [24].

## 3. Results

**Figure 1:**
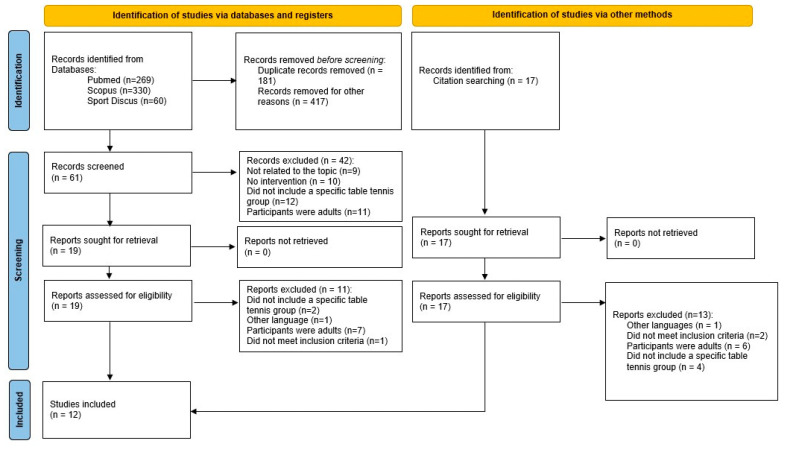
Flow diagram of the review process.

The initial search yielded a total of 659 records (PubMed n = 269, Scopus n = 330, and SportDiscus n = 60) (Figure 1). After applying the initial exclusion criteria, 181 studies were discarded due to duplication and 417 for being irrelevant to the search topic, resulting in a total of 61 studies classified as “valid (a priori)”. After a full-text review of these studies, 53 were further excluded for the following reasons: the study was not focused on children or adolescents (18 studies); the study was in a language other than Spanish or English (10 studies); the study did not have a specific experimental TT group or was not an intervention (24 studies); the study did not provide an accurate description of the data analyzed (1 study). Therefore, eight articles were selected for the review.

The main search was supplemented by a second search reviewing the citations and references of the previously selected studies, yielding a total of 17 valid studies for full-text review. After reading these, 13 articles were discarded for the following reasons: the study was not focused on children or adolescents (six studies); the study was in a language other than Spanish or English (three studies); the study did not have a specific experimental TT group (four studies). Finally, four additional studies were included for analysis (Figure 1).

### 3.1. Designs and Participants

At the conclusion of the search, a qualitative analysis was conducted to determine the typology of each study. The results included three randomized controlled trials [25,26,27], eight comparative studies [28,29,30,31,32,33,34,35], and one uncontrolled study [36].

The sample sizes ranged from 11 [36] to 135 children and adolescents [35]. The mean ages in the studies ranged from 4.60 years [26] to 13.75 years [33], with one study not specifying the mean age of the participants [30]. Among the studies, eight focused on children [25,26,27,28,29,31,32,35], two on adolescents [33,34], and two on children and adolescents [30,36].

The studies included samples of children with Attention Deficit Hyperactivity Disorder (ADHD) (n = 3; [25,27,31]), Developmental Coordination Disorder (DCD) (n = 3; [28,29,33]), Intellectual Disability (n = 2; [30,35]), Autism Spectrum Disorder (ASD) (n = 1; [32]), and typically developing children and adolescents (n= 3; [26,34,36]).

### 3.2. Characteristics and Results of the Exercise Interventions

Details of the general characteristics of the included studies and their TT interventions are provided in Tables S1–S5 for children/adolescents with ADHD, DCD, Intellectual Disability, ASD, and typical development, respectively.

#### 3.2.1. Children with ADHD

The three studies that conducted interventions in children with ADHD (Table S1) [25,27,31] had a duration of 12 weeks. The training frequency was two [25,31] or three times per week [27]. Each session lasted between 60 min [27] and 70 min [25,31]. The results of these interventions indicated that children and adolescents who participated in TT interventions improved their locomotor skills and executive functions (*p* < 0.05). Additionally, improvements were observed in graphomotor function (*p* < 0.01) [27], social behavior (*p* < 0.05) [25], and object control [31] (*p* < 0.05).

Three studies included comparison groups. In the studies by Chang et al. [27] and Pan et al. [31], the comparison groups consisted of children with ADHD who continued their usual routine. The results showed that the TT group outperformed the comparison group in graphomotor function, executive function, motor skills and object control (*p* < 0.05). In the crossover study by Pan et al. [25], it was observed that TT practice produced a longer-lasting residual exercise effect in the group that received the intervention first. Additionally, Chang et al. [27] proposed an intervention for the comparison group based on virtual TT, where no significant differences were found compared to the TT group (*p* > 0.05). One study included a comparison group consisting of children with typical development [31], and no significant differences were found between the comparison group and the TT group (*p* > 0.05).

#### 3.2.2. Children with DCD

The duration of the interventions (Table S2) ranged from 8 weeks [33] to 12 weeks [29], with a training frequency of three sessions per week in all three interventions. The duration of each session varied from 40 min [29] to 90 min [33]. All three studies concluded that TT intervention in children and adolescents with DCD improved their motor skills. Additionally, improvements were observed in inhibition capacity [28], haptic function [29], and visual perception [33].

Three studies included comparison groups. In the studies by Kim et al. [33], Tsai [28], and Tseng et al. [29], the comparison groups consisted of children with DCD who continued their usual routine. The results showed that the TT group had greater inhibition capacity, better motor skills, and greater improvement in visual perception (*p* < 0.05). One study included a comparison group consisting of children with typical development [28], where the TT group showed an inferior reaction time compared to the comparison group (*p* < 0.05).

#### 3.2.3. Children and Adolescents with Intellectual Disabilities

The duration of the two interventions (Table S3) in children with intellectual disabilities was 8 weeks [30] and 16 weeks [35]. Both interventions included three weekly sessions of 60 min each. These two studies assessed different variables, concluding improvements after the intervention such as improved visual perception [35], improved executive function [35], and improved working memory [30].

Two studies included comparison groups. In the studies by Chen et al. [35] and Sabzi et al. [30], the comparison groups consisted of children and adolescents with intellectual disabilities who continued their usual routine. The results indicated that the TT group outperformed the comparison group in visual perception, executive function, and working memory. In this context, Chen et al. [35] proposed an intervention for the comparison group based on occupational therapy, observing that the TT group had greater executive function and visual perception improvements than the previous groups.

#### 3.2.4. Children with ASD

This review identified a single study on the effects of TT in children with ASD (Table S4). The study conducted by Pan et al. [32] proposed a 12-week intervention consisting of two weekly sessions of 70 min each. The intervention reported improvements in motor skills and executive functions in children and adolescents after the intervention (*p* < 0.01). In this crossover study, it was observed that TT practice produced a longer-lasting residual exercise effect in the group that received the intervention first.

#### 3.2.5. Children and Adolescents with Typical Development

The duration of the interventions ranged from 6 weeks [36] to 1 year [34], with a training frequency varying from two times per week [36] to three to five times per week [34] (Table S5). These three studies assessed different variables, concluding improvements after the intervention such as improved gross motor skills (*p* < 0.01) [26], improved attention capacity (*p* < 0.05) [36], and improved coordination (*p* < 0.01) [34].

Two studies included comparison groups. In the studies by Chagas et al. [34] and Gu et al. [26], the comparison groups consisted of children and adolescents with typical development who continued their usual routine. The results indicated that the TT group improved coordination (*p* = 0.01) and gross motor skills (*p* < 0.05) compared to the comparison group.

### 3.3. Methodological Quality

The included RCT studies were scored as having fair [27]-to-good [25,26] quality using the PEDro scale. The prevalent flaws were the lack of concealed allocation, and that none of the studies had blinded subjects or therapists administering the exercise sessions (Table 1).

Seven out of the nine non-randomized studies were classified as high quality [28,29,30,31,32,33,35]. However, several weaknesses were noted. Of the nine studies reviewed, only three included consecutive patients [29,33,34]. Notably, Tseng et al. [29] and Tsai [28] were the sole studies that conducted an unbiased assessment of the study endpoint and Sabzi et al. [30] was the only one to carry out a prospective calculation of the study size (Table 2).

## 4. Discussion

This review primarily aimed to identify and analyse the existing scientific literature on the effects of TT in children and adolescents. A total of 12 studies were included, examining the effects of TT in children and adolescents with ADHD, DCD, ASD, intellectual disabilities, and typically developing children. Based on the extracted data, several insights can be drawn that may be useful for the studied population and professionals who work with them.

One intriguing finding was that research on the benefits of TT has concentrated more on children with special needs than on typically developing children. A plausible explanation could be that children with special needs often encounter unique physical, emotional, and social challenges, and TT can be adapted to meet these specific needs. Indeed, TT has frequently been employed as a therapeutic strategy to enhance motor proficiency and executive function, both of which can be positively influenced through exercise in this population [37].

Scahill and Schwab-Stone [38] define ADHD as a chronic mental health condition characterized by difficulty maintaining attention, impulsive behaviours, and hyperactivity. Rusca-Jordán and Cortez-Vergara [39] report that the prevalence of this disorder ranges from 2% to 12% in the paediatric population. Meggs et al. [40] propose physical exercise as a low-cost and effective treatment for reducing ADHD symptoms. In this review, TT was found to be effective in improving executive function, graphomotor function, social behaviour, motor skills, and object control. Several studies have observed similar results after the administration of different exercise programs in this population. For instance, Ziereis and Jansen [41] reported significant improvements in executive function and motor skills in a sample of children with ADHD who took part in a physical activity program consisting of one 60 min session per week that combined balance training with ball games for twelve weeks. Similar results were observed after a 10-week program that included three 45 min sessions per week involving progressive aerobic, muscular, and motor skill exercises [42]. In this line, a number of studies have also confirmed that exercise, including ball activity games, can lead to improvements in the social behaviour of children with ADHD [43]. Finally, it is worth noting that TT has led to improvements in graphomotor function, a key characteristic of ADHD that can be enhanced through motor training [44]. This suggests that TT presents new opportunities for addressing ADHD through targeted motor activities. Notably, when TT was compared with children who did not undergo the intervention, its effects were superior in the aforementioned variables.

According to Ros Cervera et al. [45], DCD is a chronic neuromotor disorder common in children, with a prevalence of 5–6% in school-aged children. It is characterized by motor coordination below the expected level for their age, affecting fine and gross motor skills. In this review, TT had positive effects on both executive function and motor skills, aligning with previous findings. For instance, a training program consisting of three 60 min sessions per week that combined fitness and agility activities for 8 weeks led to improvements in motor skills as assessed by the MABC [46]. Similar results were observed after administering a motor skill training program to children with DCD, which included 60 min sessions of skill and agility training performed three times a week for 8 weeks [47]. Notably, these studies, as well as those included in this review, proposed a frequency of three training days per week. Jane et al. [48] indicated that the effectiveness of interventions aimed at improving motor skills in children with DCD is influenced by training frequency, with a higher number of sessions per week leading to more robust results in this population.

According to the reviewed studies, TT was also effective in stimulating inhibition capacity, demonstrating similar effects to other sports modalities. For instance, a soccer training program with 50 min sessions conducted five times a week for 10 weeks led to improvements in inhibitory control in children with ADHD [49]. Notably, TT also improved haptic function and visual perception. These findings align TT with other novel therapeutic approaches that have shown positive results in these areas, such as haptic perception training [50] and Wii Fit training [51].

Finally, when TT intervention results were compared with those of children with DCD who continued their usual routines, the TT group showed better values in inhibition capacity, motor skills, and visual perception. Moreover, in this research, the effects of the program in children with DCD were compared with typically developing children. The results suggested that children with DCD who participated in the TT program had slower reaction times than typically developing children.

Luckasson et al. [52] define Intellectual Disability as having an IQ score of 70 or below, affecting 2–3% of the population regardless of society. Individuals with this disability have long-term memory difficulties and struggle to learn, with greater difficulty retaining more abstract information [53]. In the present review, TT had positive effects on this population. Chen et al. [35] found improvements in visual perception and executive function, while Sabzi et al. [30] reported improvements in working memory. Other authors, such as Fragala-Pinkham et al. [54], indicated that children with disabilities can improve their cardiorespiratory endurance after an aquatic aerobic exercise program. Giagazoglou et al. [55] demonstrated balance improvements in individuals with intellectual disabilities through a trampoline exercise program. Finally, when the results of TT interventions were compared with those of children with intellectual disabilities who continued their usual routines, the TT group showed better values in visual perception, executive function, and working memory. Additionally, in the study by Chen et al. [35], the effects of TT were compared with an occupational therapy program. The results suggested that the TT group had greater improvements in executive function and visual perception than the occupational therapy group.

Bentham et al. [1] describe ASD as a neurodevelopmental disorder with neurobiological origins, beginning in childhood, that affects social communication development and behaviour, characterized by repetitive and restricted behaviours and interests. According to the World Health Organization (WHO) [56], 1 in 100 children has autism. In this review, TT had positive effects on this population. Pan et al. [32] demonstrated the effects of TT, reporting improvements in motor skills and executive functions after the intervention. Another study by Movahedi et al. [57] observed improvements in closed motor skills through a kata program. Additionally, Nicholson et al. [58] showed that an aerobic training program improved academic performance in adolescents with ASD. Finally, the crossover study by Pan et al. [32] observed that TT practice produced a longer-lasting residual exercise effect in the group that received the intervention first.

Regarding typically developing children, this review reported improvements in gross motor skills [26], attention capacity [36], and coordination [34]. Other articles, such as the article by Van der Niet et al. [59], also reported improvements in attention capacity and other executive functions. Yasumitsu and Nogawa [60] found that a coordination exercise intervention during school recess improved coordination. Finally, when TT was compared with children who did not undergo the intervention, its effects were superior in the aforementioned variables.

Several limitations need to be acknowledged in this review. Firstly, no analysis of the methodological quality of the studies was conducted, nor was the quality of the interventions reported. Furthermore, although various conditions were found, the sample sizes for each were small. Finally, we focused on studies in English and languages we were fluent in due to resource constraints. Although evidence suggests that our approach should not significantly impact the conclusions [61,62], restricting the search to specific languages and databases may have limited the number of results found.

## 5. Conclusions

Scientific evidence focusing on the effects of TT in children and adolescents comes from studies with diverse samples, including those who are typically developing and those with various pathologies. The results indicate that practicing TT at least twice a week for a minimum of six weeks can positively impact cognitive and motor skills and improve social behavior. Furthermore, TT has been suggested as a therapeutic strategy for children with intellectual disabilities, ADHD, DCD, and ASD. To maximize its benefits, TT sessions should include hand–eye coordination exercises, group games, specific skills training with partners, and socialization activities.

## Figures and Tables

**Table 1:** Methodological quality appraisal of the included randomized controlled trials.

Study	Assessment Items										
Randomized controlled trials (PEDro scale)	1	2	3	4	5	6	7	8	9	10	Total Score
Chang et al. [27]	+	-	+	-	-	-	+	-	+	+	5/10
Gu et al. [26]	+	-	+	-	-	-	+	+	+	+	6/10
Pan et al. [25]	+	-	+	-	-	-	+	+	+	+	6/10

1 = Random allocation; 2 = concealed allocation; 3 = baseline comparability; 4 = blind subjects; 5 = blind therapists; 6 = blind assessors; 7 = adequate follow-up; 8 = intention-to-treat analysis; 9 = between-group comparisons; 10 = point estimates and variability.

**Table 2:** Methodological quality appraisal of the non-randomized studies included.

**Study**	**Assessment Items**												
Non-Randomized Studies (Methodological Index for Non-Randomized Studies, MINORS)	1	2	3	4	5	6	7	8	9	10	11	12	Total Score
Sabzi et al. [30]	2	0	2	1	0	2	2	1	2	2	2	2	18/24
Kim et al. [33]	1	1	2	2	0	2	2	0	1	2	2	2	17/24
Tseng et al. [29]	2	2	2	2	2	2	2	0	2	2	2	2	22/24
Pan et al. [31]	2	0	2	2	0	2	2	0	2	2	2	2	18/24
Chagas et al. [34]	2	2	0	2	0	2	2	0	2	2	0	2	16/24
Pan et al. [32]	2	0	2	2	0	2	2	0	2	2	2	2	18/24
Chen et al. [35]	2	0	2	2	0	2	2	0	2	2	2	2	18/24
Tsai [28]	2	0	2	2	2	2	1	0	2	2	2	2	19/24
Salici and Söyleyici [36]	2	0	2	1	0	2	2	0	-	-	-	-	7/16

1 = Clearly stated aim; 2 = inclusion of consecutive patients; 3 = prospective collection of data; 4 = endpoints appropriate to the aim of the study; 5 = unbiased assessment of the study endpoint; 6 = follow-up period appropriate to the aim of the study; 7 = loss to follow-up less than 5%; 8 = prospective calculation of the study size; 9 = adequate control group; 10 = contemporary groups; 11 = baseline equivalence of groups; 12 = adequate statistical analyses.

## Data Availability

Data are contained within the article.

## Supplementary Materials

The following supporting information can be downloaded at https://www.mdpi.com/article/10.3390/children11080963/s1. Table S1: General characteristics of the studies in children with ADHD; Table S2: General characteristics of studies in children with DCD; Table S3: General characteristics of studies in children with Intellectual Disabilities; Table S4: General characteristics of the study in children with ASD; Table S5: General characteristics of studies with typically developing children.
