# Supplementary material for: Benefits of Table Tennis for Children and Adolescents: A Narrative Review
Source: Children (Basel). 2024 Aug 10;11(8):963. doi: 10.3390/children11080963 (PMC11353217; doi:10.3390/children11080963)
Supplement: Supplementary file 1 [file children-11-00963-s001.zip › children-3129580-supplementary.pdf]

Supplementary Table S1. General characteristics of the studies in children with ADHD.

First autor (Year); design	Participants	Intervention	Outcomes (assessment tool)	Results
Shao-Hsia Chang (2022); Randomized	Sample Size: 48 children with ADHD<br>Completed the program: 48<br>Dropouts and cause: 0<br><br>TTG:<br>n=16 (boys: 13, girls: 3)<br>Mean age ± SD: 8.31 ± 1.30 years<br>Right-handed: 13<br>Left-handed: 3<br><br>CG1:<br>n=16 (boys: 13, girls: 3)<br>Mean age ± SD: 8.38 ± 1.20 years<br>Right-handed: 14<br>Left-handed: 2<br><br>CG2:<br>n=16 (boys: 13, girls: 3)<br>Mean age ± SD: 8.38 ± 1.31 years<br>Right-handed: 14<br>Left-handed: 2	Duration: 12 weeks<br>Frequency: 3 sessions/week Session duration: 60 minutes<br><br>TTG:<br>Structured TT program including: a) basic exercises; b) attention and cognitive stimulation exercises; c) eye-hand coordination exercises.<br><br>CG1:<br>Virtual TT program on Nintendo Wii consisting of 3 mini-games: Target TT, Exciting TT, and Matching TT. Frequency: 3 times/week, 1 hour of exercise.<br><br>CG2:<br>No training	Executive Function:<br>-Stroop Color-Word Test<br>-The Wisconsin Card Sorting Test<br><br>Graphomotor Function:<br>-Graphomotor Test	Intragroup:<br>-Improvement in all characteristics of the graphomotor test in TTG ($p<0.01$)<br>-Improvement in all characteristics of the graphomotor test in CG1 ($p<0.01$)<br>-Decrease in the total number of errors (WCST) in TTG ($p<0.01$)<br>-Decrease in perseverative response (WCST) in TTG ($p<0.01$)<br>-Improvement in TTG in the Color-Word Test ($p<0.01$)<br>-Improvement in CG1 in the Color-Word Test ($p<0.05$)<br><br>Intergroup:<br>-Higher performance in the graphomotor test in TTG and CG1 compared to CG2<br>-Greater improvement in the Stroop Color-Word Test in TTG and CG1 compared to CG2 ($p=0.017$)

Chien-Yu Pan (2019); comparative	Sample size: 60 children Completed the program: 60 Dropouts and cause: 0<br>TTG: n=15 children with ADHD (6 without medication and 9 with)<br>Mean age ± SD: 9.08 ± 1.43 years<br>Height: 134.35 ± 11.55 cm<br>Weight: 32.30 ± 10.61 kg<br>BMI: 17.55 ± 4.00 kg/m^2<br><br>GM1: n=15 children with ADHD (6 without medication and 9 with)<br>Mean age ± SD: 8.90 ± 1.66 years<br>Height: 134.00 ± 9.97 cm<br>Weight: 32.89 ± 9.60 kg<br>BMI: 18.14 ± 4.24 kg/m^2<br><br>CG2: n=30 typical development children<br>Mean age ± SD: 9.14 ± 1.54 years<br>Height: 135.52 ± 12.27 cm<br>Weight: 35.14 ± 11.21 kg<br>BMI: 18.78 ± 4.00 kg/m^2	Duration: 12 weeks<br>Frequency: 2 sessions/week<br>Session duration: 70 minutes<br><br>TTG: TT program structured as: warm-up, basic skills, executive function training, group games, and cool-down.<br><br>CG1: No change in lifestyle.<br>CG2: No change in lifestyle.	Locomotor skills and object control:<br>-Gross Motor Development–2 Test<br>-Stroop Color-Word Test<br>-Wisconsin Card Sorting Test	Intragroup:<br>-Locomotor skills improved in TTG (+5.13) (p<0.01)<br>-Object control improved in TTG (+5.20) (p<0.01)<br>-Locomotor skills improved in CG1 (+1.27) (p<0.01)<br>-Locomotor skills improved in CG2 (+4.63) (p<0.01)<br>-Object control improved in CG2 (+2.23) (p<0.01)<br>-Stroop Color-Word condition improved in TTG (+8.63) (p<0.01)<br>-Stroop Color-Word condition improved in CG2 (+4.30) (p<0.01)<br>-Improvement in the total number of correct responses (WCST) in TTG (+10.93) (p<0.01)<br>-Decrease in perseverative errors (WCST) in TTG (-10.63) (p<0.01)<br>-Decrease in perseverative errors (WCST) in CG2 (-3.17) (p<0.01)<br>-Improvement in the number of categories completed (WCST) in TTG (+1.60) (p<0.01)<br>-Improvement in the number of categories completed (WCST) in CG1 (+0.67) (p<0.05)<br>-Improvement in the number of categories completed (WCST) in CG2 (+0.68) (p<0.01)<br><br>Intergroup:<br>-Improvement in locomotor skills (+3.33) and object control (+4.67) in TTG compared to CG1<br>-Improvement in locomotor skills (+3.63) and object control (+3.63) in CG2 compared to CG1<br>-TTG (+8.20) and CG2 (+8.13) improved in the Stroop Color-Word condition compared to CG1<br>-Improvement in the total number of correct responses (WCST) in TTG (+9.80) compared to CG1

Chien-Yu_Pan (2016); randomized	Sample size: 32 children<br>Completed the program: 32<br>Dropouts and cause: 0<br><br>TTG1:<br>n=16 children with ADHD (9 of them on medication)<br>Mean age ± SD: 8.93 ± 1.49 years<br>Weight: 30.68 ± 8.71 kg<br>Height: 132.86 ± 11.64 cm<br>BMI: 17.11 ± 2.87 kg/m^2<br><br>TTG2:<br>n=16 children with ADHD (9 of them on medication)<br>Mean age ± SD: 8.87 ± 1.56 years<br>Weight: 32.62 ± 9.34 kg<br>Height: 133.73 ± 9.69 cm<br>BMI: 18.07 ± 4.10 kg/m^2	Duration: 12 weeks Frequency: 2 sessions/week<br>Session duration: 70 minutes<br><br>TT program focused on improving: motor skills, social behaviors, and cognitive functions. Each session will consist of the following phases: warm-up, motor skills training, executive function training, group games, and cool-down.<br><br>TTG_1: Performed the intervention and subsequently acts as CG.<br><br>TTG_2: Starts as CG and subsequently performs the intervention.	Motor skills:<br>-Bruininks-Oseretsky Test 2<br>Social behavior:<br>-Chinese version of the Child Behavior Checklist<br>Neuropsychological measures:<br>-Stroop Color-Word	**Intragroup:**<br>Phase 1 (after the first 12 weeks):<br>-TTG_1 improved strength and agility scores (BOT-2) ($p<0.01$)<br>-TTG_1 improved the total motor composite (BOT-2) ($p<0.01$)<br>-Decrease in social problems scores (CBCL) in TTG_1 ($p<0.01$)<br>-Decrease in attention problems scores (CBCL) in TTG_1 ($p<0.01$)<br>-Decrease in aggressive behaviors scores (CBCL) in TTG_1 ($p<0.01$)<br>-Decrease in externalizing problems scores (CBCL) in TTG_1 ($p<0.01$)<br>-Decrease in total social problems (CBCL) in TTG_1 ($p<0.01$)<br>-Improvement in Stroop Color-Word in TTG_1 ($p<0.01$)<br><br>Phase 2 (From week 12 to 24):<br>-TTG_2 improved strength and agility scores (BOT-2) ($p<0.01$)<br>-TTG_2 improved the total motor composite (BOT-2) ($p<0.01$)<br>-Decrease in social problems scores (CBCL) in TTG_2 ($p<0.01$)<br>-Decrease in attention problems scores (CBCL) in TTG_2 ($p<0.01$)<br>-Decrease in aggressive behaviors scores (CBCL) in TTG_2 ($p<0.01$)<br>-Decrease in externalizing problems scores (CBCL) in TTG_2 ($p<0.01$)<br>-Decrease in total social problems (CBCL) in TTG_2 ($p<0.01$)<br>-Improvement in Stroop Color-Word in TTG_2 ($p<0.01$)<br><br>**Intergroup:** TTG_1 had a longer-lasting residual exercise effect than TTG_2.

BMI: body mass index; CG: control group; SD: standard deviation; TTG: table tennis group.

Supplementary Table S2. General characteristics of studies in children with DCD.

First autor (Year); design	Participants	Intervention	Outcomes (assessment tool)	Results
Chia-Liang Tsai (2009); comparative	Sample size: 57 children<br>Completed the program: Not specified<br>Dropouts and cause: Not specified<br><br>TTG:<br>n=14 children with DCD<br>Mean age ± SD: 114.38 ± 4.37 months<br>Weight: 30.68 ± 8.71 kg<br>Height: 132.86 ± 11.64 cm<br>BMI: 17.11 ± 2.87 kg/m^2<br><br>CG1:<br>n=14 children with DCD<br>Mean age ± SD: 113.64 ± 3.48 months<br><br>CG2:<br>n=29 typical development children<br>Mean age ± SD: Not specified	Duration: 10 weeks<br>Frequency: 3 sessions/week<br>Session duration: 50 minutes<br><br>TTG: TT program with the following structure: warm-up, TT training, TT with a partner, and cool-down. The program increases in technical difficulty as the weeks progress.<br><br>CG1: Performed usual physical education activities and does not engage in any new sports outside of school.<br><br>CG2: Performed usual physical education activities and does not engage in any new sports outside of school.	Motor skills:<br>- Movement Assessment Battery for Children-2<br><br>Behavioral inhibition capacity:<br>- Endogenous model of visuospatial attention	**Intragroup:**<br>-TTG improved motor skills (p=0.002)<br>-Improvement in the inhibitory control strength difference in the TTG group (p=0.005)<br>-Reaction time improved in all 3 groups<br><br>**Intergroup:**<br>-TTG and CG1 performed worse than CG2 in the motor skills test (p=0.01) (p<0.001)<br>-TTG performed better than CG1 in the motor skills test (p=0.002)<br>-CG1 has worse behavioral inhibition capacity than TTG and CG2<br>-TTG and CG1 have worse reaction times than CG2
Yu-Ting Tseng (2023); comparative	Sample size: 20 children with probable DCD<br>Completed the program: 20<br>Dropouts and cause: 0<br><br>TTG:<br>n=10 (3 boys and 7 girls)<br>Mean age ± SD: 9.50 ± 0.70<br>All right-handed<br><br>CG1:<br>n=10 (3 boys and 7 girls)<br>Mean age ± SD: 9.2 ± 0.42<br>All right-handed	Duration: 12 weeks<br>Frequency: 3 sessions/week<br>Session duration: 40 minutes<br><br>TTG: TT program with the following structure: warm-up, TT training, and cool-down. The goal of the program was to improve haptic function and upper limb motor skills. The TT training includes: balancing with the ball, wall hits, shadow swings, serves, forehand hits, and comprehensive practice.<br><br>CG1: Continue with their daily life, refraining from racket sports and playing musical instruments.	Haptic function:<br>- Haptic system<br><br>Motor skills:<br>- Movement Assessment Battery for Children-2	**Intragroup:**<br>-Improvement in haptic sensitivity in the TTG<br>-Improvement in haptic detection threshold in the TTG<br>-Improvement in the aiming and catching time (AC in the MABC-2 test) in the TTG<br>-Improvement in the manual dexterity time (MD in the MABC-2 test) in the TTG<br>-Improvement in the manual dexterity time (MD in the MABC-2 test) in the CG1<br><br>**Intergroup:** None
Dongmin Kim (2024); comparative	Sample size: 31 children with DCD<br>Completed the program: 31<br>Dropouts and cause: 0.<br><br>TTG:	Duration: 8 weeks<br>Frequency: 3 sessions/week<br>Session duration: 90 minutes<br><br>TTG: TT program structured in: warm-up,	Visual Perception:<br>- Korean Developmental Test of Visual Perception-Adolescent<br><br>Motor Skill:	Intragroup:<br>-Improvement of TTG in the K-DTVP-A test (p<0.001)<br>-Improvement of TTG in the BOTMP-2 test on motor skill development (p<0.001)

	n=16 (14 boys and 2 girls)<br>Mean age ± SD: 13.75 ± 1.07<br>Weight: 76.03 ± 17.43 kg<br>Height: 167.39 ± 7.20 cm<br>DCDQ-K: 33.42 ± 8.05<br>BOTMP-2: 7.82% ± 3.30<br><br>CG1:<br>n=15 (12 boys and 3 girls)<br>Mean age ± SD: 13.73 ± 1.03 y<br>Weight: 64.83 ± 15.14 kg<br>Height: 166.60 ± 5.22 cm<br>DCDQ-K: 34.52 ± 9.04<br>BOTMP-2: 9.02% ± 3.17	skill training (serve, hit, reception), match, and cool-down. This program also introduces a task-oriented approach methodology to facilitate learning and improvement in children. This methodology has been previously validated and works with children with DCD.<br><br>CG1: Does not participate in the TT intervention. No other conditions.	- Bruininks-Oseretsky Test of Motor Proficiency-2<br>- Developmental Coordination Disorder Questionnaire	Intergroup:<br>-Greater improvement of TTG in the K-DTVP-A test compared to CG1<br>-Greater improvement of TTG in the BOTMP-2 test on motor skill development compared to CG1

BMI: body mass index; CG: control group; SD: standard deviation; TTG: table tennis group

Supplementary Table S3. General characteristics of studies in children with Intellectual Disabilities.

First autor (Year); design	Participants	Intervention	Outcomes (assessment tool)	Results
Ming-De Chen (2015); comparative	Sample size: 135 children<br>Completed the program: 132<br>Dropouts and cause: 3. Not specified<br><br>TTG:<br>n=45 (24 boys and 21 girls) (22 with mild intellectual disability and 23 borderline)<br>Mean age ± standard deviation: 10.60 ± 3.60 y<br>Weight: 47.20 ± 10.20 kg<br><br>CG1:<br>n=46 (26 boys and 20 girls) (22 with mild intellectual disability and 24 borderline)<br>Mean age ± standard deviation: 10.90 ± 3.90<br>Weight: 46.80 ± 9.90 kg<br><br>CG2:<br>n=41 (23 boys and 18 girls) (20 with mild intellectual disability and 21 borderline)<br>Mean age ± standard deviation: 10.70 ± 4.00 y<br>Weight: 48.20 ± 9.30 kg	Duration: 16 weeks<br>Frequency: 3 sessions/week<br>Session duration: 60 minutes<br><br>TTG:<br>TT program with 4 main components: serves, forehand hits, backhand hits, and continuous hits with 50 balls at different speeds. The sessions will increase in difficulty and intensity as the weeks progress.<br><br>CG1:<br>Standard occupational therapy program including neurodevelopmental, perceptual-motor, and sensory integration treatment approaches<br><br>CG2:<br>No change in lifestyle.	Visual Perception:<br>- Test of Visual Perceptual Skill–third edition<br><br>Executive Function:<br>- Stroop Color-Word<br>- Wisconsin Card-Sorting Test 64-card version	Intragroup:<br>-TTG improved in 7 of the 8 items of the TVPS-3 test, 4 of the 6 items of the WCST-64, and 2 of the 3 items of the Stroop test (Cohen's d-value > 0.80)<br>-CG1 improved in 7 of the 8 items of the TVPS-3 test, 4 of the 6 items of the WCST-64, and 2 of the 3 items of the Stroop test (Cohen's d-value > 0.50)<br><br>Intergroup:<br>-TTG and CG1 outperformed CG2 in all tests<br>-TTG outperformed CG1 in the subtests of: spatial relations, form constancy, sequential memory, and figure-ground<br>-In the WCST-64, TTG performed better in all subtests than CG1<br>-TTG performed better than CG1 in the Stroop color and Color-Word tests
Amir Hamzeh Sabzi (2023); comparative	Sample size: 30 girls with intellectual disabilities aged between 9-15 years<br>Completed the program: 30<br>Dropouts and cause: 0<br><br>TTG:<br>n=15<br>Mean age ± standard deviation: Not specified<br><br>CG1:<br>n=15<br>Mean age ± standard deviation: Not specified	Duration: 8 weeks<br>Frequency: 3 sessions/week<br>Session duration: 60 minutes<br><br>TTG: TT program structured in: warm-up, skill training, group games, and cool-down. The technical skills learned advanced in difficulty as the weeks progressed.<br><br>CG1: They do not engage in any activity beyond their usual daily routine.	Working Memory:<br>- Daneman and Carpenter's Working Memory Test	Intragroup:<br>-Improvement in working memory in TTG (p<0.001)<br><br>Intergroup:<br>-The improvement in working memory in TTG was greater than in CG1

BMI: body mass index; CG: control group; SD: standard deviation; TTG: table tennis group.

Supplementary Table S4. General characteristics of the study in children with ASD.

First autor (Year); design	Participants	Intervention	Outcomes (assessment tool)	Results
Chien-Yu Pan (2017); comparative	Sample size: 22 children<br>Completed the program: 22<br>Dropouts and cause: 0<br><br>TTG:<br>n=11 children (6 with autism and 5 with Asperger syndrome) (3 of them also with ADHD)<br>Mean age ± SD: 9.68 ± 1.61<br>Height: 137.88 ± 12.25 cm<br>Weight: 35.93 ± 11.45<br>BMI: 18.56 ± 3.73 kg/m^2<br>ABCT score: 13.45 ± 6.39<br>SRS-2 score: 113.82 ± 19.34<br><br>TTG2:<br>n=11 children (6 with autism and 5 with Asperger syndrome) (3 of them also with ADHD)<br>Mean age ± SD: 8.49 ± 1.76<br>Height: 134.55 ± 12.99 cm<br>Weight: 32.00 ± 12.47<br>BMI: 17.02 ± 3.66 kg/m^2<br>ABCT score: 14.09 ± 7.23<br>SRS-2 score: 111.64 ± 19.33	Duration: 12 weeks<br>Frequency: 2 sessions/week<br>Session duration: 70 minutes<br><br>All sessions will be structured as: Warm-up, motor skills, executive function motor skills, group games, and cool-down.<br>During the first 4 weeks, basic exercises with TT balls will be performed, and in the following 8 weeks, more complex exercises and games will be added.<br><br>TTG1: Performed the intervention and subsequently acts as GC.<br><br>TTG2: Starts as GC and subsequently performs the intervention.K.	Motor skills:<br>- Bruininks–Oseretsky Test of Motor Proficiency-2<br><br>Executive functions:<br>- Wisconsin Card Sorting Test	Intragroup:<br>-Improvement in manual coordination (BOT-2) in TTG1 (+9.73) (p<0.01)<br>-Improvement in general coordination (BOT-2) in TTG1 (+10.00) (p<0.01)<br>-Improvement in strength and agility (BOT-2) in TTG1 (+13.27) (p<0.01)<br>-Improvement in total motor composite (BOT-2) in TTG1 (+10.36) (p<0.01)<br>-Improvement in manual coordination (BOT-2) in TTG2 (+5.91) (p<0.01)<br>-Improvement in general coordination (BOT-2) in TTG2 (+7.73) (p<0.01)<br>-Improvement in strength and agility (BOT-2) in TTG2 (+9.27) (p<0.01)<br>-Improvement in total motor composite (BOT-2) in TTG2 (+11.73) (p<0.01)<br>-Improvement in the total number of correct responses (WCST) in TTG1 (+9.09) (p<0.01)<br>-Improvement in conceptual level response (WCST) in TTG1 (+21.18) (p<0.01)<br>-Decrease in perseverative response (WCST) in TTG1 (-10.64) (p<0.01)<br>-Improvement in the total number of correct responses (WCST) in TTG2 (+5.91) (p<0.01)<br>-Improvement in conceptual level response (WCST) in TTG2 (+12.09) (p<0.01)<br><br>Intergroup:<br>-TTG1 had a longer residual effect than TTG2

BMI: body mass index; CG: control group; SD: standard deviation; TTG: table tennis group.

Supplementary Table S5. General characteristics of studies with typicall development children.

First autor (Year); design	Participants	Intervention	Outcomes (assessment tool)	Results
Ying Gu (2021); paired randomized	Sample size: 104 children<br>Completed the program: 104<br>Dropouts and cause: 0<br><br>TTG:<br>n=52 (25 boys and 27 girls)<br>Mean age ± SD: 55.190 ± 8.702<br>Height: 107.171 ± 7.649 cm<br>Weight: 17.504 ± 3.432<br><br>CG1:<br>n=52 (25 boys and 27 girls)<br>Mean age ± SD: 55.190 ± 8.997<br>Height: 107.235 ± 7.717 cm<br>Weight: 17.529 ± 2.845	Duration: 12 weeks<br>Frequency: 3 times per week<br>Session duration: 50 minutes<br><br>TTG: TT program that progresses through the following phases over the weeks: getting to know the ball, feeling the ball, imitation, technical strokes, and relaxation.<br>In these phases, participants will be introduced to TT and will end up learning the technical strokes.<br><br>CG1: Participated in the usual physical education classes mandated by the State (China).	Gross motor skills:<br>- Test of Gross Motor Development-2	Children:<br>Intragroup:<br>-Improvement in gross motor skills in the TTG ($p<0.001$)<br>-Improvement in locomotion subtest in TTG ($p<0.01$)<br>-Improvement in object manipulation subtest in TTG ($p<0.01$)<br><br>Girls:<br>Intragroup:<br>-Improvement in gross motor skills in TTG ($p<0.001$)<br>TTG improvement in locomotion subtest compared to CG1 ($p<0.001$)<br>-Improvement in object manipulation subtest in TTG ($p<0.001$)<br><br>Boys/Girls<br>Integroup:<br>-TTG improves gross motor skills compared to CG1 ($p<0.05$)<br>-TTG improves in locomotion subtest compared to CG1 ($p<0.05$)<br>-TTG improves in object manipulation subtest compared to CG1 ($p<0.05$)
Olcay Salici (2020); non-controlled	Sample size: 11 children (8 boys and 3 girls)<br>Completed the program: 11<br>Dropouts: 0<br><br>TTG:<br>n=11<br>Mean age ± SD: 9.82 ± 2.23	Duration: 6 weeks<br>Frequency: 2 times per week<br>Session duration: Not specified<br><br>TTG: TT program in which the basic forehand and backhand rally techniques are taught, with the goal of achieving the maximum number of exchanges without errors.	Attention Capacity:<br>- Bourdon Attention Test	Improvement in the attention test in the number of marked letters ($p<0.05$)
Daniel V. Chagas (2018); comparative	Sample size: 31 children<br>Completed the program: 31<br>Dropout and reason: 0	Duration: 1 year<br>Frequency: 3-5 times per week<br>Session duration: 30-40 minutes	Coordination Capacity:<br>- Körperkoordinationstest für Kinder (KTK)	Intragroup:<br>-Improvement in motor coordination values (KTK) in TTG ($p<0.0001$)<br>-Improvement in motor coordination values (KTK) in

	TTG:<br>n=17 (11 boys and 6 girls)<br>Mean age ± SD: 13.30 ± 0.50 years<br>Weight: 43.00 ± 8.7 kg<br>Height: 1.55 ± 0.10 m<br><br>CG1: 17 (6 boys and 11 girls)<br>Mean age ± SD: 13.00 ± 0.40 years<br>Weight: 52.50 ± 16.10 kg<br>Height: 1.57 ± 0.1 m	Between the sixth and seventh month, there was a 45-day break where TTG did not practice TT since it was practiced at school.<br><br>TTG: Recreational TT program that was unstructured and based on play (matches)<br><br>CG1: Participated in regular physical education classes and does not start any sport.		CG1 ($p<0.0001$)<br><br>Intergroup:<br>-The TTG group shows greater improvement in coordination levels (KTK) compared to CG1 ($p=0.01$)

BMI: body mass index; CG: control group; SD: standard deviation; TTG: table tennis group.
